# A New Species of *Vetubrachypsectra* from Mid-Cretaceous Amber of Northern Myanmar (Coleoptera: Brachypsectridae)

**DOI:** 10.3390/insects13020122

**Published:** 2022-01-25

**Authors:** Yan-Da Li, Robin Kundrata, Tian-Quan Qu, Di-Ying Huang, Chen-Yang Cai

**Affiliations:** 1 State Key Laboratory of Palaeobiology and Stratigraphy, Nanjing Institute of Geology and Palaeontology, Center for Excellence in Life and Palaeoenvironment, Chinese Academy of Sciences, Nanjing 210008, China; ydli@nigpas.ac.cn (Y.-D.L.); dyhuang@nigpas.ac.cn (D.-Y.H.); 2 Department of Zoology, Faculty of Science, Palacky University, 77146 Olomouc, Czech Republic; robin.kundrata@upol.cz; 3 State Key Laboratory of Biogeology and Environmental Geology, China University of Geosciences, Wuhan 430078, China; qtq@cug.edu.cn; 4 School of Earth Sciences, University of Bristol, Life Sciences Building, Tyndall Avenue, Bristol BS8 1TQ, UK

**Keywords:** Elateroidea, *Brachypsectridae*, sexual dimorphism, Burmese amber

## Abstract

**Simple Summary:**

Brachypsectridae is a small family in the superfamily Elateroidea, with only two extant and two extinct genera known based on adults. Here, we figure and describe a new brachypsectrid species from the mid-Cretaceous amber of northern Myanmar, based on an exceptionally well-preserved female specimen. Although sexual dimorphism is common in Brachypsectridae, this specimen is recognized as a new species, rather than a female of the previously reported *V. burmitica*, primarily based on its different pedicel–scape attachment.

**Abstract:**

A new species, *Vetubrachypsectra huchengi* Li, Kundrata & Cai sp. nov., is described from mid-Cretaceous Burmese amber on the basis of a single adult female. The species is assigned to genus *Vetubrachypsectra* Qu & Cai based on its serrate antennae, long maxillary palps, presence of tibial spurs, and elytra without clear striae. *Vetubrachypsectra huchengi* differs distinctly from *V. burmitica* Qu & Cai, the only other species in the genus, in having the pedicel apically attached to the scape. Some other differences between the female of *V. huchengi* and the male of *V. burmitica* include less serrate antennae, a broader pronotal disc, a broader scutellar shield and smaller tibial spurs. However, at least some of these characters can be considered sexually dimorphic.

## 1. Introduction

Brachypsectridae is a small family in the superfamily Elateroidea. The whole family was known from a single extant genus, *Brachypsectra* LeConte, until the discovery of *Asiopsectra* Kovalev & Kirejtshuk in 2016 [1]. *Brachypsectra* currently comprises seven extant species and have been reported from various sites around the world, including the southwestern part of North America, the Dominican Republic, Turkey, Iran, Cyprus, India, and Singapore [2,3,4,5,6,7], while two species of *Asiopsectra* are only known from Tajikistan and Iran based on a single male specimen each [1]. Adults of brachypsectrids generally share a similar habitus with other hard-bodied elateroid taxa [8], though without a functional prothoracic “clicking mechanism” [9]. Larvae of brachypsectrids, in contrast, are quite peculiar in having branched lateral lobes on the thorax and abdomen [2,3,4,7,8,10,11,12]. Two recent large-scale phylogenetic studies, using morphological (implied weighted parsimony) [13] and molecular (Bayesian) [14] data, respectively, suggested Brachypsectridae as sister to a group comprised of Throscidae, Eucnemidae, and Cerophytidae, although in the latter study [14], analysis under maximum likelihood found Brachypsectridae sister to the “higher Elateroidea” *sensu* Kundrata et al. [15].

The easily distinguishable larval fossils of Brachypsectridae have been reported from Miocene Dominican amber [3,4,16], Eocene Baltic amber [12,16], and mid-Cretaceous Burmese amber [12,17]. An adult fossil of *Brachypsectra* is also known from Dominican amber [4]. Recently, two fossil genera of Brachypsectridae were established based on adults found in Burmese amber. *Hongipsectra* Tihelka et al. is distinctive in having 11-segmented bilamellate antennae in males and a posterior pronotal margin with an M-shaped medial notch [18]. *Vetubrachypsectra* Qu & Cai is morphologically similar to *Brachypsectra*, although it can be separated from the latter based on the longer maxillary palpomere 3, presence of tibial spurs, and parameres without a hook [19]. Genus *Cretopsectra* was erected based on a larva in Burmese amber [17], which is problematic because it might belong to some previously described species, as noted by Haug et al. [12]. In the current study, we report an additional new species of *Vetubrachypsectra* based on a single female from Burmese amber, and re-examine the holotype of *V. burmitica* using confocal microscopy.

## 2. Materials and Methods

The Burmese amber specimens studied herein, i.e., holotypes of both species of *Vetubrachypsectra*, originated from amber mines near Noije Bum (26°20′ N, 96°36′ E), Hukawng Valley, Kachin State, northern Myanmar. Both specimens are deposited in the Nanjing Institute of Geology and Palaeontology (NIGP), Chinese Academy of Sciences, Nanjing, China. The amber piece containing the new species was trimmed with a small table saw, ground with emery paper of different grit sizes, and finally polished with polishing powder.

Photographs under incident light were taken with a Zeiss Discovery V20 stereo microscope. Widefield fluorescence images were captured with a Zeiss Axio Imager 2 light microscope combined with a fluorescence imaging system. Confocal images were obtained with a Zeiss LSM710 confocal laser scanning microscope, using the 488 nm (Argon; for *V. huchengi* sp. nov.) or 561 nm (DPSS 561-10; for *V. burmitica*) laser excitation lines [20]. Fluorescence images could illustrate the structures better than brightfield ones, as the fluorescence emitted around the border between the inclusion and amber matrix clearly shows the boundary of structures. In confocal microscopy, the out-of-focus background fluorescence produced by the amber matrix is further blocked, leading to a higher signal-to-noise ratio [20]. Images under incident light and widefield fluorescence were stacked in Helicon Focus 7.0.2 or Zerene Stacker 1.04. Confocal images were stacked with Helicon Focus 7.0.2 and Adobe Photoshop CC. Images were further processed in Adobe Photoshop CC to adjust brightness and contrast.

This published work and the nomenclatural act have been registered in ZooBank, the official registry of Zoological Nomenclature. The LSID for this publication is urn:lsid:zoobank.org:pub:CC211990-2ABF-40EF-B419-61A7507BEAEF.

## 3. Systematic Palaeontology

Order Coleoptera Linnaeus, 1758

Suborder Polyphaga Emery, 1886

Superfamily Elateroidea Latreille, 1804

Family Brachypsectridae Horn, 1881

Genus *Vetubrachypsectra* Qu & Cai, 2019

**Figure 1:**
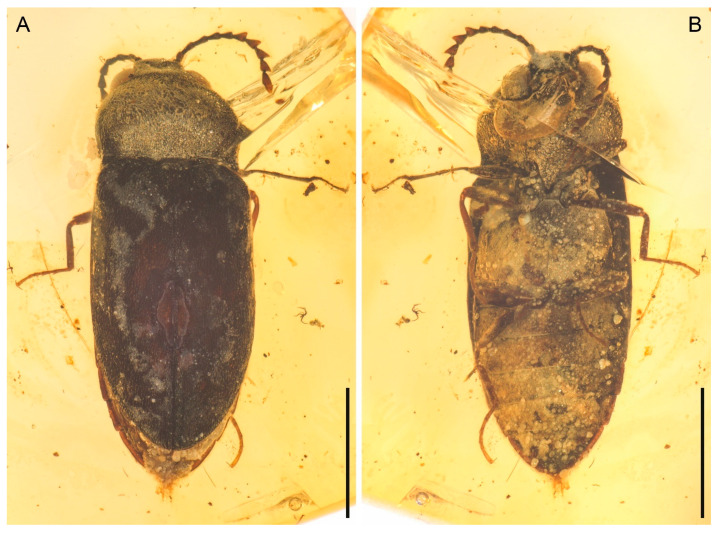
General habitus of *Vetubrachypsectra huchengi* Li, Kundrata & Cai sp. nov., holotype, NIGP177984, under incident light. (**A**) Dorsal view. (**B**) Ventral view. Scale bars: 1.5 mm.

**Figure 2:**
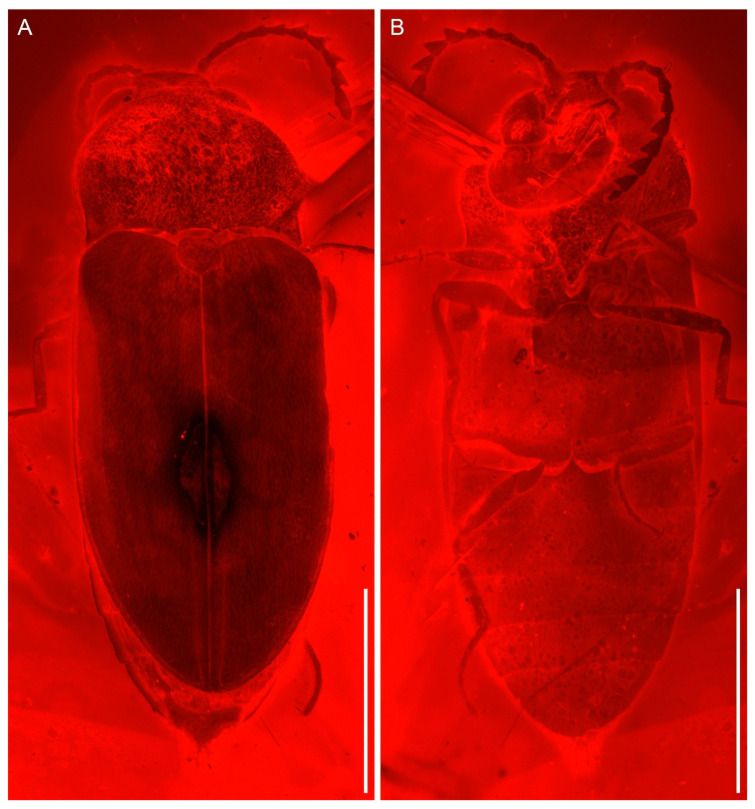
General habitus of *Vetubrachypsectra huchengi* Li, Kundrata & Cai sp. nov., holotype, NIGP177984, under widefield fluorescence. (**A**) Dorsal view. (**B**) Ventral view. Scale bars: 1.5 mm.

**Figure 3:**
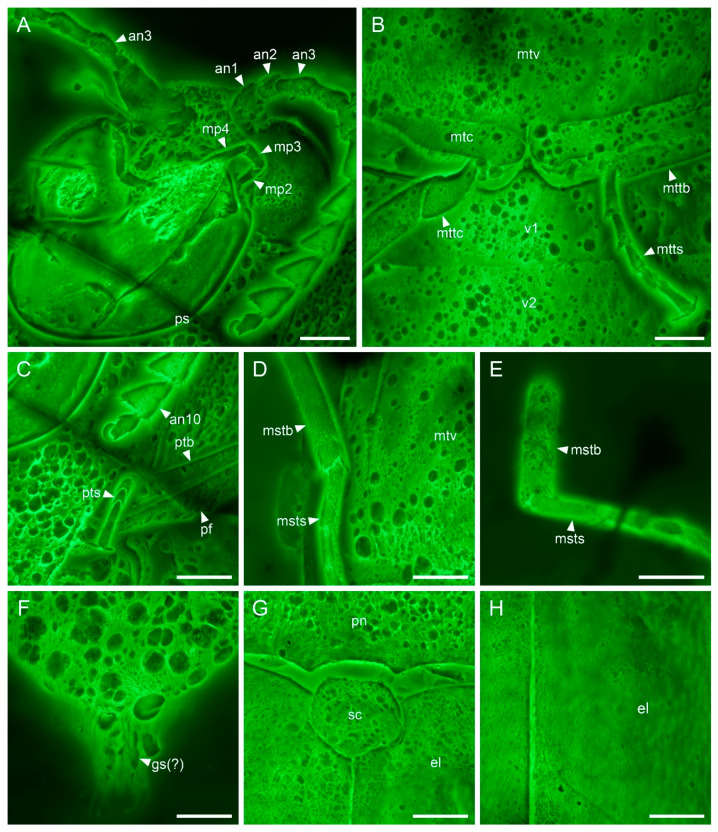
Details of *Vetubrachypsectra huchengi* Li, Kundrata & Cai sp. nov., holotype, NIGP177984, under confocal microscopy. (**A**) Head, ventral view. (**B**) Hind legs. (**C**) Fore leg. (**D**,**E**) Mid legs. (**F**) Ovipositor, ventral view. (**G**) Scutellum, dorsal view. (**H**) Elytra, dorsal view. Abbreviations: an1–10, antennomeres 1–10; el, elytron; gs, gonostylus; mp2–4, maxillary palpomeres 2–4; mstb, mesotibia; msts, mesotarsus; mtc, metacoxa; mttb, metatibia; mttc, metatrochanter; mtts, metatarsus; mtv, metaventrite; pf, profemur; pn, pronotum; ps, prosternum; ptb, protibia; pts, protarsus; sc, scutellum; v1,2, ventrites 1,2. Scale bars: 200 μm.

***Vetubrachypsectra huchengi***  **Li, Kundrata & Cai sp. nov.**

(Figure 1, Figure 2 and Figure 3)

**LSID.** urn:lsid:zoobank.org:act:B87EE252-E6FD-44AA-92B3-995BCF2EC551

**Etymology.** This species is named after Mr. Cheng Hu, who kindly donated many fossils for our research.

**Type material.** Holotype, NIGP177984, adult female (NIGP).

**Locality and horizon.** Amber mine located near Noije Bum Village, Tanai Township, Myitkyina District, Kachin State, Myanmar; unnamed horizon, mid-Cretaceous, Upper Albian to Lower Cenomanian.

**Diagnosis. Adult female.** Antennae 11-segmented, distinctly serrate from antennomeres 7 to 10; pedicel apically attached to scape. Maxillary palps long; palpomere 3 distinctly longer than wide. Posterior pronotal margin without M-shaped medial notch. Prosternal process triangular, comparatively wide basally. Anterior scutellar margin without medial notch. Elytra without row of punctures or clear striae. Metaventral discrimen only visible near the posterior end. Tibial spurs 2-2-2.

**Description. Adult female.** Body elongate and flattened, about 4.9 mm long, 1.9 mm wide.

Head hypognathous, slightly declined, deeply inserted into prothorax. Eyes large, globular, strongly protruding. Antennal insertions concealed from above. Frontoclypeal suture absent. Antennae elongate, 11-segmented; pedicel (antennomere 2) shorter and more slender than scape (antennomere 1), apically attached to scape; antennomeres 3 and 4 elongate; antennomeres 5 and 6 shorter than 3 and 4, slightly expanded apically; antennomeres 7–10 distinctly asymmetrically expanded apically (serrate); antennomere 11 fusiform, seemingly with a subapical notch. Maxillary palps elongate; palpomeres 2–4 distinctly longer than wide.

Pronotal disc broad, about 1.5 times as wide as length along middle, with distinct lateral carinae; anterior angles broadly rounded; posterior angles acute, produced laterally and posteriorly, embracing elytral bases; lateral sides sinuate; posterior edge trisinuate. Prosternum in front of coxae broad. Prosternal process triangular, comparatively wide basally, acute apically, fitting into mesoventral cavity.

Scutellar shield comparatively broad, wider than long, subpentagonal. Elytra about 1.8 times as long as greatest combined width and 3.0 times as long as pronotum, not completely covering abdomen; sides subparallel in basal half, tapering apically; surface with small punctures not arranged in clear rows; surface with only inconspicuous striae. Mesoventrite short, broad; mesoventral cavity with well-developed walls. Metaventrite broad; discrimen only visible near the posterior end; transverse (katepisternal) suture absent. Metacoxae strongly transverse, extending laterally to meet elytra; metacoxal plates complete, narrow, subparallel in medial half, then constricted laterally.

Legs slender and simple. Trochanters moderately elongate; trochanterofemoral joints on fore and mid legs slightly oblique, those on hind legs strongly oblique. Tibial spurs 2-2-2, small. Tarsal formula 5-5-5; tarsomeres simple, without ventral lobe. Pretarsal claws simple.

Abdomen with five ventrites. Ventrites 1–4 subequal in length, ventrite 5 broadly rounded at apex.

**Remark.** This species is placed to *Vetubrachypsectra* based on the elongate maxillary palpomere 3, presence of tibial spurs, and elytra without clear striae [19].

## 4. Discussion

Currently, there are two extant and two extinct genera (with adults known) in Brachypsectridae [1,4,18,19]. The extinct genus *Vetubrachypsectra* differs from the other three genera in having the combination of unipectinate/serrate antennae, an elongate maxillary palpomere 3, the presence of tibial spurs, and elytra without clearly defined striae.

**Figure 4:**
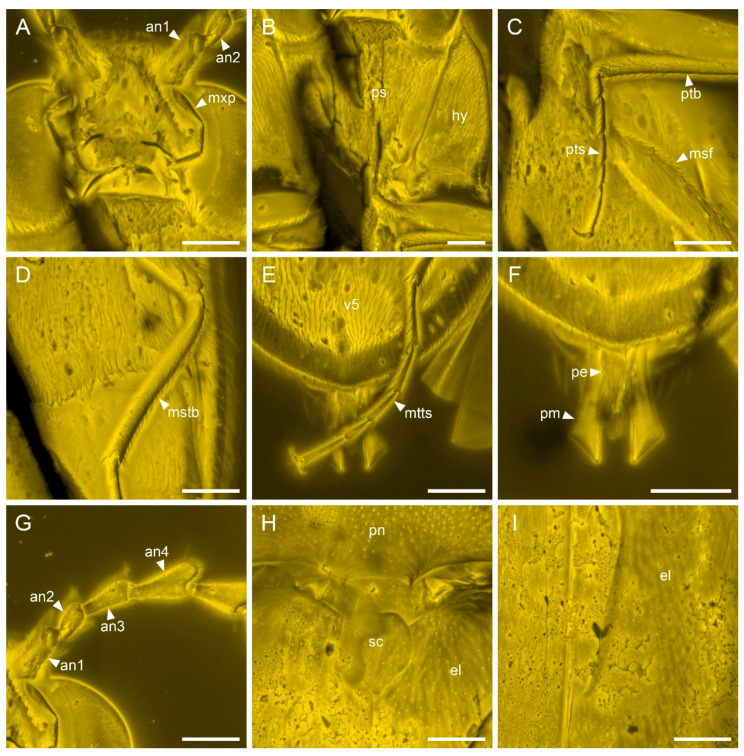
Details of *Vetubrachypsectra burmitica* Qu & Cai, holotype, NIGP170326, under confocal microscopy. (**A**) Head, ventral view. (**B**) Prothorax, ventral view. (**C**) Fore leg. (**D**) Mid leg. (**E**) Hind leg. (**F**) Aedeagus, ventral view. (**G**) Antennal base, ventral view. (**H**) Scutellum, dorsal view. (**I**) Elytra, dorsal view. Abbreviations: an1–4, antennomeres 1–4; el, elytron; hy, hypomeron; msf, mesofemur; mstb, mesotibia; mtts, metatarsus; mxp, maxillary palp; pe, penis; pm, paramere; pn, pronotum; ps, prosternum; ptb, protibia; pts, protarsus; v5, ventrite 5. Scale bars: 200 μm.

Compared to the previously published male of *V. burmitica* Qu & Cai (Figure 4) [19], the female of *V. huchengi* sp. nov. exhibits several different characters (Figure 1, Figure 2 and Figure 3). The pronotum is only moderately convex dorsally, about 1.3 times as wide as long, and with less rounded sides in *V. burmitica*, while it is more robust and convex dorsally, 1.5 times as wide as long, and with clearly rounded sides in *V. huchengi* sp. nov. In *V. burmitica*, the antenna is distinctly pectinate in antennomeres 4–10, while it is distinctly serrate only in antennomeres 7–10 in *V. huchengi* sp. nov. The posterior margin of the outer portion of metacoxal plates in *V. burmitica* is more or less straight, but it might be gradually rounded in *V. huchengi* sp. nov. (at least as seen in the right metacoxal plate). Additionally, *V. huchengi* sp. nov. has a distinctly broader scutellar shield (wider than long vs. longer than wide) and less well-developed tibial spurs.

Modern members of *Brachypsectra* display some sexual dimorphism in body size and shape, with females being generally larger and broader, and having less pectinate/serrate antennae and a somewhat anteriorly inflated pronotal disc [4,6]. Similar dimorphism has also been suggested for the fossil genus *Hongipsectra* from Burmese amber [18]. The less prominently serrate (and not pectinate) antennae, more transverse and dorsally convex pronotal disc, broader scutellar shield and smaller tibial spurs of *V. huchengi* sp. nov. (all or only some of them) may actually represent sexually dimorphic characters as well. However, the specimen of *V. huchengi* has the pedicel apically attached to the scape (Figure 3A), while *V. burmitica* has the pedicel attached to the scape subapically (Figure 4A,G) [19]. Since both specimens are well preserved and there are no differences between right and left antenna within the same specimen, this character cannot be classified as an artifact due to the pressures during the formation of the amber piece. However, such a character is not known as sexually dimorphic in any Brachypsectridae known so far. Actually, the subapical attachment of pedicel to scape is used for diagnoses of higher-ranked taxa (Cerophytidae and Eucnemidae) rather than species. Thus, the attachment of pedicel is very unlikely to be a sexually dimorphic character, and we suggest *V. huchengi* sp. nov. should represent a separate species, rather than the female of *V. burmitica*.

## Data Availability

The original confocal data are available on Zenodo repository (doi:10.5281/zenodo.5871683).

## References

[1] Kovalev A.V., Kirejtshuk A.G. (2016). *Asiopsectra* gen. n., a second genus of the family Brachypsectridae (Coleoptera, Elateroidea) from the Palaearctic Region. Insect Syst. Evol..

[2] Blair K. (1930). *Brachypsectra*, Lec.—The solution of an entomological enigma. Trans. R. Entomol. Soc. Lond..

[3] Woodruff R.E. (2002). A new species of the beetle genus *Brachypsectra* from the Dominican Republic, with fossil connections (Coleoptera: Brachypsectridae). Insecta Mundi.

[4] Costa C., Vanin S.A., Lawrence J.F., Ide S., Branham M.A. (2006). Review of the family Brachypsectridae (Coleoptera: Elateroidea). Ann. Entomol. Soc. Am..

[5] Hájek J. (2010). Brachypsectra kadleci sp. nov. from western Iran—the first Palaearctic member of the family Brachypsectridae (Insecta: Coleoptera: Elateriformia). Ann. Zool..

[6] Petrzelkova I., Makris C., Kundrata R. (2017). The genus *Brachypsectra* LeConte, 1874 (Coleoptera: Brachypsectridae) in the Palaearctic Region. Eur. J. Taxon..

[7] Lawrence J.F., Monteith G.B., Reid C.A. (2020). A new *Brachypsectra* LeConte from Australia (Coleoptera: Brachypsectridae) with comparative notes on adults and larvae. Pap. Avulsos Zool..

[8] Fleenor S.B., Taber S.W. (1999). Review of *Brachypsectra* LeConte with a new record of the Texas beetle (*B. fulva* LeConte; Coleoptera: Brachypsectridae). Coleopt. Bull..

[9] Young D.K., Arnett R.H., Thomas M.C., Skelley P.E., Frank J.H. (2002). Brachypsectridae Bøving and Craighead 1931. American Beetles. Vol. 2. Polyphaga: Scarabaeoidea through Curculionoidea.

[10] Barber H.S. (1905). Illustrations of an undetermined coleopterous larva. Proc. Entomol. Soc. Wash..

[11] Ferris G. (1927). Notes on an entomological enigma. Can. Entomol..

[12] Haug J.T., Zippel A., Haug G.T., Hoffeins C., Hoffeins H.-W., Hammel J.U., Baranov V., Haug C. (2021). Texas beetle larvae (Brachypsectridae)—The last 100 million years reviewed. Palaeodiversity.

[13] Lawrence J.F., Ślipiski A., Seago A.E., Thayer M.K., Newton A.F., Marvaldi A.E. (2011). Phylogeny of the Coleoptera based on morphological characters of adults and larvae. Ann. Zool..

[14] Mckenna D.D., Wild A.L., Kanda K., Bellamy C.L., Beutel R.G., Caterino M.S., Farnum C.W., Hawks D.C., Ivie M.A., Jameson M.L. (2015). The beetle tree of life reveals that Coleoptera survived end-Permian mass extinction to diversify during the Cretaceous terrestrial revolution. Syst. Entomol..

[15] Kundrata R., Bocakova M., Bocak L. (2014). The comprehensive phylogeny of the superfamily Elateroidea (Coleoptera: Elateriformia). Mol. Phylogenet. Evol..

[16] Klausnitzer B. (2009). Bemerkungen zu rezenten und fossilen Larven (bernstein) der Gattung *Brachypsectra* LeConte (Coleoptera, Brachypsectridae). Contrib. Nat. Hist..

[17] Zhao X., Zhao X., Jarzembowski E., Tian Y., Chen L. (2020). The first record of brachypsectrid larva from mid-Cretaceous Burmese amber (Coleoptera: Polyphaga). Cretac. Res..

[18] Tihelka E., Huang D., Cai C. (2019). Diverse Texas beetles (Coleoptera: Elateroidea: Brachypsectridae) in mid-Cretaceous Burmese amber: Sexual dimorphism and palaeoecology. Palaeoentomology.

[19] Qu T., Yin Z., Huang D., Cai C. (2020). First Mesozoic brachypsectrid beetles in mid-Cretaceous amber from northern Myanmar (Coleoptera: Elateroidea: Brachypsectridae). Cretac. Res..

[20] Fu Y.-Z., Li Y.-D., Su Y.-T., Cai C.-Y., Huang D.-Y. (2021). Application of confocal laser scanning microscopy to the study of amber bioinclusions. Palaeoentomology.

